# Failure Prediction of Lithium Disilicate and Composition-Gradient Multilayered Zirconia Occlusal Veneers: A Fractographic and Theoretical Analysis

**DOI:** 10.3390/ma18184287

**Published:** 2025-09-12

**Authors:** Lea S. Prott, Petra C. Gierthmuehlen, Markus B. Blatz, Yu Zhang

**Affiliations:** 1 Department of Preventive and Restorative Sciences, Penn Dental Medicine, University of Pennsylvania, Philadelphia, PA 19104, USA; mblatz@upenn.edu (M.B.B.); yz21@upenn.edu (Y.Z.); 2 Department of Prosthodontics, Medical Faculty and University Hospital Düsseldorf, Heinrich-Heine-University, 40225 Düsseldorf, Germany; petra.gierthmuehlen@med.uni-duesseldorf.de

**Keywords:** occlusal veneer, ceramic thickness, fatigue fracture, fracture mechanics, zirconia, lithium disilicate

## Abstract

This in vitro study aimed to evaluate the fatigue behavior of occlusal veneers (OVs) made of lithium disilicate and composition-gradient multilayered zirconia at different thicknesses, incorporating both experimental and theoretical analyses to predict long-term performance. Seventy-two OVs with ceramic layer thicknesses of 0.5 mm, 1.0 mm, and 1.5 mm were fabricated and adhesively bonded to dentin analog composite abutments. All specimens underwent thermomechanical fatigue testing, involving cyclic loading (49 N, 1.6 Hz, 1.2 million cycles) and thermocycling (5–55 °C), simulating five years of clinical function. Fracture patterns were analyzed using light microscopy and scanning electron microscopy. A fatigue lifetime model based on plate-on-foundation theory and slow crack growth was applied to estimate cycles to radial failure. No complete fractures or debonding occurred. However, 50% of 0.5 mm zirconia OVs developed flexural radial cracks from the intaglio surface, while all lithium disilicate and zirconia veneers ≥1.0 mm remained intact. Theoretical predictions closely matched the experimental outcomes, indicating that 0.5 mm zirconia performance aligned with the lower-bound fatigue estimates for 5Y-PSZ. Results suggest that lithium disilicate offers superior fatigue resistance at minimal thickness, while thin zirconia is prone to subsurface cracking. A minimum thickness of 0.7 mm is recommended for zirconia-based OVs.

## 1. Introduction

In recent years, occlusal veneers (OVs) have emerged as a minimally invasive alternative to traditional onlays, characterized by a non-retentive preparation design and reduced layer thickness [1]. Digital workflows, including intraoral scanning, virtual mock-ups and chairside milling enable the fabrication of ultrathin restorations that deliver excellent marginal adaptation and structural accuracy while conserving tooth tissue [2]. Previous in vitro studies have shown that OVs with a thickness of 0.7 mm or less can be successfully used to restore the occlusal vertical dimension in patients with severe tooth wear [3]. Due to its favorable esthetic and mechanical properties (adequate flexural strength (400–600 MPa), an elastic modulus similar to enamel, wear-friendly behavior and reliable bondability), lithium disilicate glass ceramic is widely used in the fabrication of OVs [4,5,6]. IPS e.max CAD (Ivoclar Vivadent, Schaan, Liechtenstein) consists of a glassy matrix mainly composed of SiO_2_, Li_2_O, P_2_O_5_, ZrO_2_, ZnO, K_2_O and Al_2_O_3_ with lithium disilicate (Li_2_Si_2_O_5_) as the predominant crystalline phase [7,8]. A recently published systematic review found that CAD/CAM fabricated composite OVs exhibit fatigue and fracture resistance comparable of lithium disilicate veneers, offering a less brittle, dentin-like alternative [9]. However, the only available clinical study reported 3-year survival rates of 100% for OVs fabricated from lithium disilicate (IPS e.max CAD, Ivoclar Vivadent) compared to 84.7% for those made of resin–matrix ceramics (Lava Ultimate, 3M Espe) [10]. These findings emphasize the importance of selecting an appropriate restorative material.

Dental zirconia ceramics have evolved from a largely uniform material with a single composition and homogeneous microstructure into more advanced, often graded structures, characterized by variations in yttria and alumina content, as well as differences in grain type and size [11]. After mechanical and esthetic modifications of the first- and second-generation of zirconia (3Y-TZP (3 mol% yttria stabilized zirconia polycrystals) with or without 0.25 wt.% alumina sintering additives, respectively), highly translucent third-generation zirconia 4Y-PSZ and 5Y-PSZ (4 mol% and 5 mol% yttria partially stabilized zirconia, respectively) gained significant interest for the fabrication of OVs [12]. With increasing yttria content, the proportion of cubic grains increased (~10–20% in 3Y-TZP, ~40% in 4Y-PSZ and ~50% in 5Y-PSZ) [13]. This resulted in enhanced translucency, but at the expense of flexural strength, which decreased from 1000–1400 MPa in 3Y-TZP to 700–1000 MPa in 4Y-PSZ and 400–800 MPa in 5Y-PSZ [14].

In 2015, multilayered fourth-generation zirconia systems were firstly introduced to mimic the shade gradient of natural teeth through pigmentation; hence, they provide a color gradient but a uniform composition with no difference in flexural strength between the enamel and dentin layers [15,16]. More recently, fifth-generation zirconia has been developed by integrating different zirconia compositions within a single blank to combine high flexural strength in the dentin region with increased translucency in the incisal or occlusal areas [14]. Previous studies have shown that different layers of these fifth-generation multilayered zirconia possess distinct compositions, thereby affecting their mechanical and optical properties and, consequently, the fracture resistance of the final restoration [17,18]. Critical flaws introduced during machining or post-processing handling are located in different areas of the blank depending on the fabrication method, while the type of zirconia (3Y, 4Y or 5Y) at the fracture origin also plays a decisive role in fracture resistance [14]. Consequently, true gradient multilayered zirconia materials introduce new challenges to researchers, dental technicians and clinicians. Moreover, predicting clinical outcomes in terms of fracture resistance is particularly complex, as intraoral conditions expose restorations to liquid media, temperature fluctuations that cause repeated thermal expansion and contraction, and cyclic multi-axial loading that generate complex stress fields [19,20]. Manufacturers’ recommendations provide important guidance; however, they are largely based on extensive laboratory testing with only limited clinical evidence available at the time of product introduction. Therefore, restoration design and positioning within the blank must adhere to the manufacturer’s recommendations, while also being guided by a fundamental understanding of the selected zirconia material [21]. Clinicians should be aware that any modifications beyond these guidelines fall outside the validated indications.

Fractographic analysis, which investigates fracture surfaces to determine crack origin, direction and failure mode, is increasingly used in dentistry and medicine to access structural integrity, detect material defects and study fatigue behavior [22]. When combined with textural analysis, it can provide additional insights into surface microstructure and wear patterns. These methods can also complement or, in some cases, serve as alternatives to some forms of imaging, including 3D imaging using Cone Beam Computed Tomography (CBCT), intraoral scanning and traditional 2D X-rays, especially when 3D imaging is unavailable, impractical, or as an adjunct for surface-level assessment in post-implantation follow-up [23]. The physical mechanisms of fatigue in ceramic restorative materials, especially in restorations cemented to tooth structures under cyclic masticatory loading, have not yet been thoroughly investigated [24]. In particular, evidence on the fatigue behavior of fifth-generation, composition-gradient multilayered OVs is scarce and warrants further research [25]. Therefore, this study aimed to evaluate the thermomechanical fatigue behavior and fracture mechanisms of OVs fabricated from gradient multilayered zirconia and lithium disilicate, bonded to and supported by a dentin analog substrate.

## 2. Materials and Methods

### 2.1. Specimen Preparation

**Figure 1:**
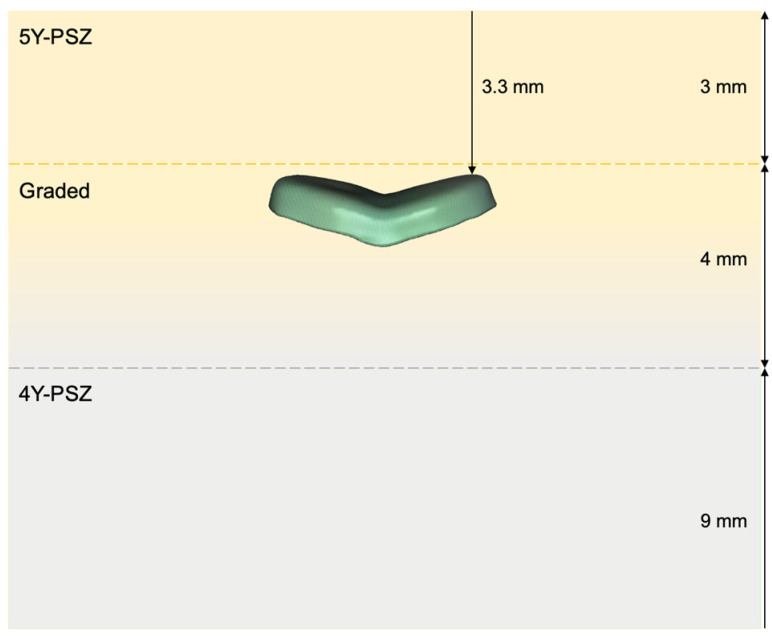
Occlusal veneer (thickness *d* = 0.5 mm, 1 mm, and 1.5 mm) positioned within the transition layer of the gradient multilayer zirconia blank, with a 3.3 mm distance from the upper border of the blank to the top surface of the veneer.

Seventy-two OV specimens were fabricated from two ceramic materials: IPS e.max CAD LT A2, Ivoclar Vivadent) and composition-gradient multilayered zirconia (Z, IPS e.max ZirCAD Prime Esthetic A2, Ivoclar Vivadent), each in three different thicknesses (0.5 mm, 1.0 mm, 1.5 mm). A maxillary molar typodont model (Frasaco-model, Frasaco, Tettnang, Germany) was used to create standardized, non-retentive master dies, which were replicated using a resin composite (Filtek Z100, 3M ESPE, Neuss, Germany) to simulate dentin analog abutments, with an elastic modulus similar to dentin (~18 GPa). This material is a visible-light activated, radiopaque composite with a BIS-GMA/TEGDMA resin matrix. The filler consists of zirconia/silica with an inorganic filler loading of 66 vol% and particle size range of 0.01–3.5 µm [26]. OVs were designed digitally (Exocad Dental CAD, Exocad, Darmstadt, Germany) and milled (PrograMill PM7, Ivoclar Vivadent) from lithium disilicate blocks or zirconia blanks, with zirconia specimens positioned within the transition layer of the multilayered blank (Figure 1). This alignment should reflect clinical CAD/CAM positioning, where the occlusal surface is located in the more translucent 5Y-PSZ for esthetics, while the adjacent 4Y-PSZ layer contributes to higher fracture resistance. IPS e.max CAD blocks were milled (PrograMill PM7 v98.130, Ivoclar Vivadent) in the pre-crystallized (blue) stage, with a milling time of 38 min. The restorations were subsequently subjected to crystallization firing (Programat EP 3010, P161, Ivoclar Vivadent) to achieve their final mechanical properties and esthetics. Similarly, IPS e.max ZirCAD Prime Esthetic was processed in the pre-sintered state, allowing efficient machining (milling time: 11 min), followed by high-temperature sintering (Programat S2, P3, Ivoclar Vivadent) to obtain its final strength and translucency. Surface treatments were applied according to the manufacturer’s instructions [7,27]. For adhesive cementation, a dual-curing resin cement composite (Variolink Esthetic DC, Ivoclar Vivadent) was used, consisting of a urethane dimethacrylate-based monomer matrix with ytterbium trifluoride and spheroidal mixed oxide fillers. The filler loading is ~38 vol%, with particle sizes ranging from 0.04–0.2 µm (mean 0.1 µm) [28]. To simulate five years of clinical use [29,30,31], all specimens underwent thermomechanical fatigue testing, involving cyclic loading (49 N, 1.6 Hz, 1.2 million cycles) and thermocycling (5–55 °C, 120 s dwell time) in a chewing simulator (CS 4.8 professional line, SD Mechatronik, Germany), with periodic inspection for any signs of failure. Additional details of the methodology can be found elsewhere [32].

### 2.2. Fractographic Analysis

For failure analysis, a polarized light microscope (AxioZoom V.16, Zeiss, Oberkochen, Germany) was used to determine fracture origin and failure modes. Additionally, the fracture surfaces of the specimens were examined using an environmental scanning electron microscope (Quanta 600 ESEM, Thermo Fisher Scientific, Waltham, MA, USA). To assess surface involvement, a backscattered electron (BSE) detector was employed. BSE imaging provides material (atomic number, Z) contrast, where heavier elements appear brighter. In this mode, cracks appear as very dark, sharp lines due to the high contrast between the material and vacuum, with the latter appearing nearly black. The smooth, even coloring of the abraded area suggests a uniform material composition and indicates no detectable damage.

### 2.3. Theoretical Analysis

**Figure 2:**
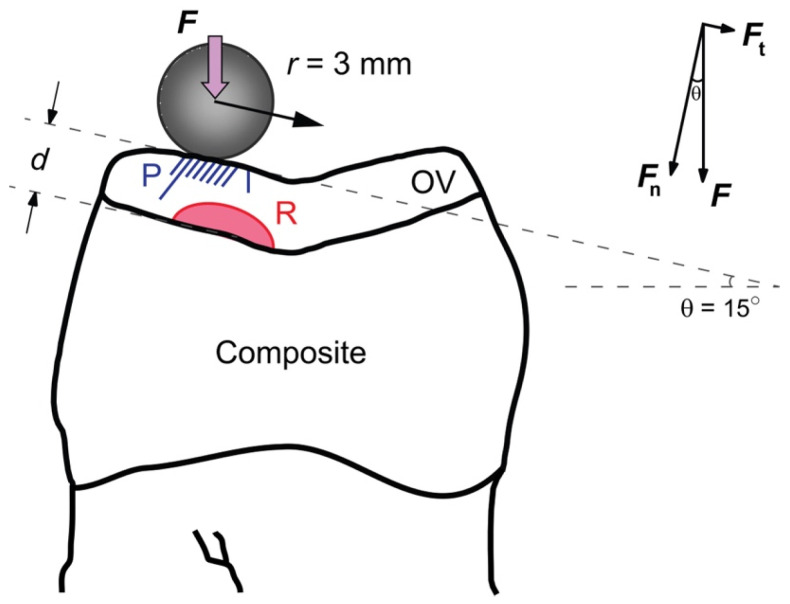
Schematic drawing of load application on occlusal veneer, based on the geometry of a standardized first maxillary molar typodont model. Average thickness *d*: 0.5 mm, 1 mm, or 1.5 mm; P: Partial cone cracks; R: Radial cracks.

When a ceramic OV is adhesively bonded to and supported by a compliant dentin abutment analog, two types of fractures may occur due to masticatory contact with the opposing dentition: near-field occlusal surface partial cone cracks induced by frictional sliding contact and far-field cementation surface flexural radial cracks induced by ceramic flexure [33] (Figure 2). The initial occlusal surface partial cone crack sizes are typically in the micrometer range, due to a steep decline in stress concentration beneath the Hertzian contact area [33]. Their propagation is driven by both chemical moist-assisted slow crack growth and mechanical hydraulic pumping fatigue [33]. Therefore, in tough zirconia and glass-ceramic materials, the propagation rate of partial cone cracks is relatively slow, and these cracks are mainly responsible for cusp chip fractures in ceramic restorations [33]. The flexural radial cracks, however, appear as pop-ins on the millimeter scale, thus immediately posing a serious threat of catastrophic failure, especially in thin restorations such as OVs. Here, we focus on the onset of cementation flexural radial fractures in lithium disilicate and zirconia OVs adhesively bonded to a dentin analog substrate under the influence of cyclic fatigue loading, as supported by our current experimental fractographic studies.

In axial loading on the inner incline of a molar cusp, the applied load ***F*** can be decomposed into a normal load ***F*_n_** and a tangential load ***F*_t_** (Figure 2). Here, only the normal load component ***F*_n_** = ***F*** cos *θ* contributes to the flexural radial fracture at the cementation surface.

***F*_n_** = ***F*** cos *θ* (1)

where ***F*** = 49 N and *θ* = 15° for the current OV designs under the given loading configuration (Figure 2). This yields ***F***_n_ = 47.3 N.

The critical load (***F*_nR_**) required to initiate a radial fracture from the ceramic intaglio surface was determined using the plate-on-foundation theory, where the ceramic OV acts as a plate supported by a cement/dentin analog foundation. The mathematical model consists of three equations [34,35]:

(2) $$F_{nR}=\frac{B\sigma d^{2}}{log(\frac{CE}{E*})}$$

where *E* is the elastic modulus of the ceramic, zirconia (=210 GPa) and lithium disilicate (=95 GPa) [36]; *d* is the thickness of the ceramic; and *σ* is the flexural strength of the ceramic. For lithium disilicate, *σ* = 462 ± 34 MPa (measured in the authors’ lab using a total of 10 samples) [37]. For zirconia, since the OV is fabricated in the graded zone and positioned close to the 5Y-PSZ layer, the substantial 5Y-PSZ content would render its flexural strength similar to that of 5Y-PSZ: *σ* = 411 ± 107 (measured in the authors’ lab using a total of 10 samples). *C* (≈1) and *B* (=1.35) are the dimensionless constants [38]; E* represents the effective modulus of the adhesive cement/dentin analog (Z100) layer, derived from contact mechanics principles [34,39,40]:

(3) $$E*={E_{c}(\frac{E_{s}}{E_{c}})}^{L}$$

where *E*_s_ and *E*_c_ are the moduli of cement (7.9 GPa, as reported in a scientific document from Ivoclar) and dentin-like substrate (18.6 GPa) [41], respectively. *L* is a dimensionless function determined experimentally and defined by Equation (4) [34]:

(4) $$\;L=\exp\left\{{-\left[\alpha +\beta \log\left(\frac{h}{d}\right)\right]}^{\gamma }\right\}$$

where α=1.18, β=0.33, γ=3.13 [34]; and *h* denotes the cement thickness, measured in cross-sectioned specimens (~40 µm).

However, under fatigue loading, the starting flaw is subject to moisture-assisted slow crack growth prior to instability. This phenomenon is exacerbated by the applied stress intensity and can be described by:

(5) $$\sigma_{F}/\sigma_{0}=(t_{0}/t_{F}{)}^{1/N}$$

where *σ*_0_ and *t*_0_ are reference parameters related to short-term tests, i.e., the intrinsic flexural strength measured by fast fracture. The time to fracture *t_F_* is given by *t_F_* = *n*/*f*, where *n* is the number of cycles to failure and *f* is the loading frequency (1.6 Hz). *N* is the velocity exponent of slow crack growth. For a ceramic layer bonded to and supported by a compliant substrate, such values for yttria-stabilized zirconia and lithium disilicate have been previously determined as: *N* = 25 ± 2 and *N* = 20 ± 3, respectively [42].

## 3. Results

### 3.1. Fatigue Exposure

**Figure 3:**
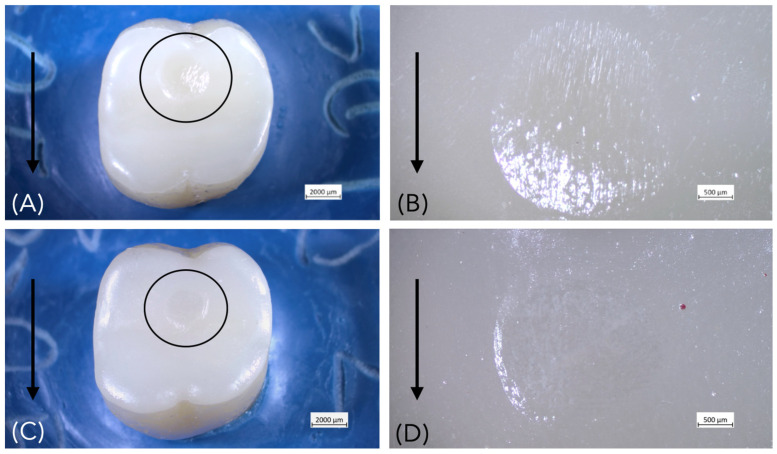
Occlusal veneer restorations following fatigue loading exhibit superficial wear facets (circled) resulting from contact with the antagonist. The bold arrow indicates the direction of sliding during fatigue. (**A**,**B**) Lithium disilicate, 0.5 mm thickness; (**C**,**D**) Zirconia, 0.5 mm thickness.

**Figure 4:**
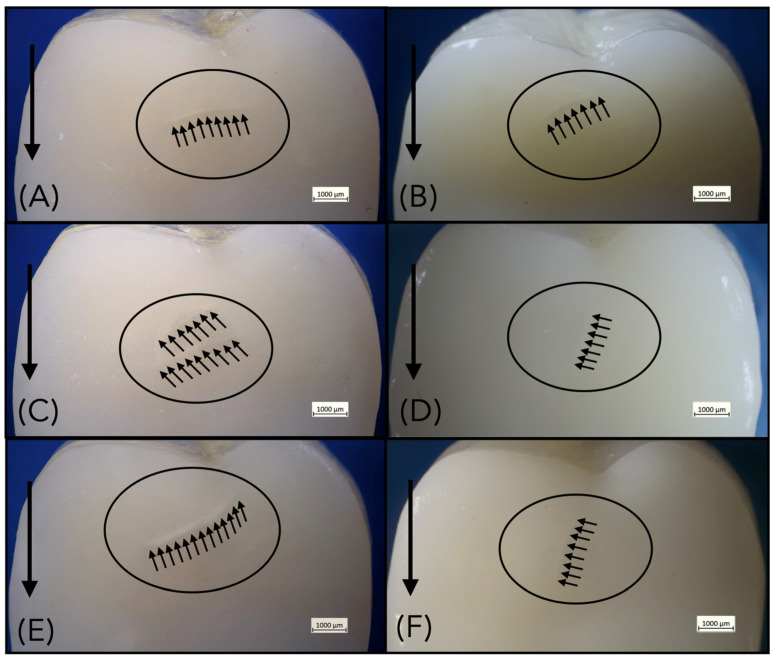
(**A**–**F**). 0.5 mm-thick zirconia restorations (group Z-0.5) after chewing simulation. Six out of 12 specimens exhibited cracks (indicated by small arrows) and wear facets (circled). The bold arrow indicates the direction of sliding during fatigue loading.

**Figure 5:**
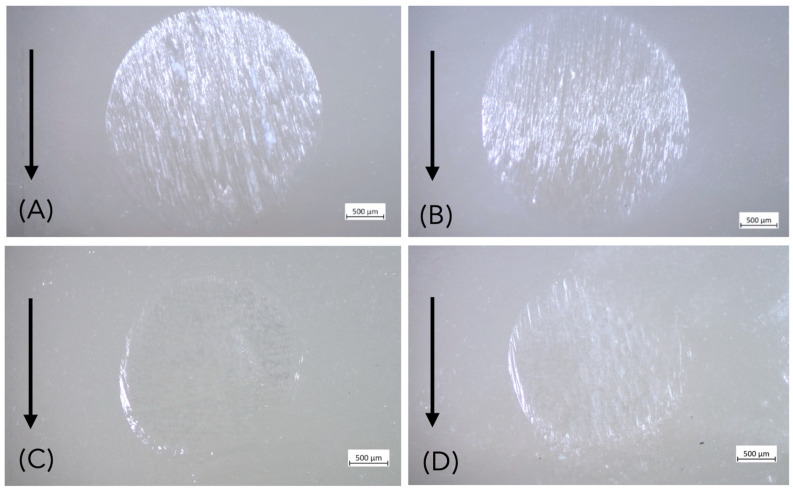
Representative specimens after chewing simulation (**A**) Lithium disilicate, 1.0 mm thickness, (**B**) Lithium disilicate, 1.5 mm thickness, (**C**) Zirconia, 1.0 mm thickness, (**D**) Zirconia, 1.5 mm thickness. All specimens exhibited wear facets but showed no cracks or fracture features. The bold arrow indicates the direction of sliding during fatigue loading.

**Figure 6:**
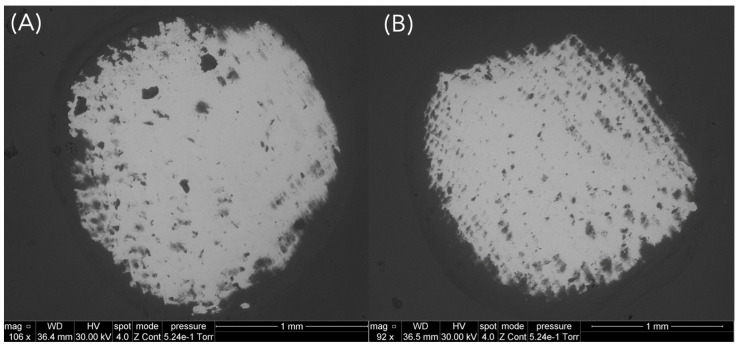
Representative SEM pictures in backscattered electron mode showing samples from group Z-0.5 with wear facets and radial cracks at intaglio surface, indicating the absence of cracks on the outer surface. (**A**) Zirconia, 0.5 mm thickness, specimen 9; (**B**) Zirconia, 0.5 mm thickness, specimen 11.

All restorations exhibited wear scars after fatigue testing, located in the area between the mesio- and distopalatal cusps due to the lateral sliding movements of the steatite ball during cyclic mechanical loading (Figure 3). After fatigue exposure, no debonding or catastrophic ceramic fractures occurred. However, half of the specimens (6/12) of group Z-0.5 (after 95,107 cycles, 95,296 cycles, 381,053 cycles, 630,042 cycles, 656,903 cycles and 840,513 cycles) developed cracks after chewing simulation (Figure 4). All specimens of lithium disilicate and thicker groups of zirconia (Z-1.0, Z-1.5) remained intact (Figure 5). Failure analysis identified cracks of group Z-0.5 as flexural radial cracks starting from the intaglio cementation surface beneath the contact point. However, despite the fact that these radial cracks are over a millimeter long (which is large relative to the thickness of the veneers), they did not propagate to the occlusal contact surface (Figure 6). Parts of these results have been published elsewhere [32].

### 3.2. Calculations

**Figure 7:**
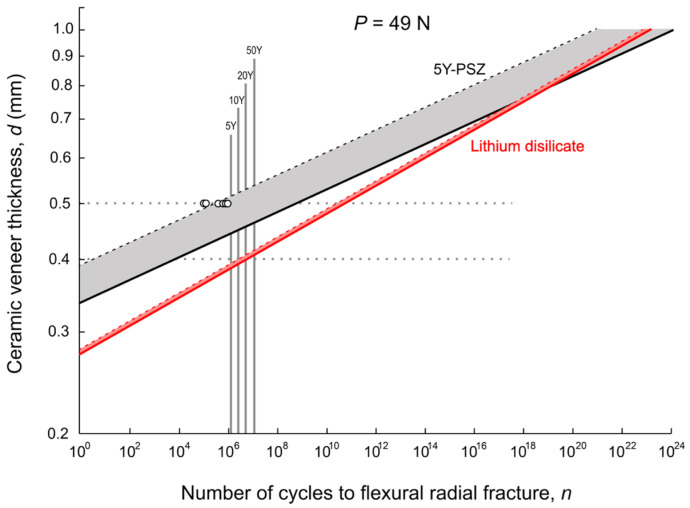
Theoretical prediction of the critical load for radial fracture as a function of the number of cycles for ceramic veneers of varying thicknesses bonded to and supported by a dentin analog composite. The shaded gray (5Y-PSZ) and red (lithium disilicate) areas represent the predicted lifetime (in number of cycles), bounded by the lower 10% (dashed lines) and mean (solid lines) *N* values for zirconia (*N*_lower_ = 23; *N*_mean_ = 25) and lithium disilicate (*N*_lower_ =17; *N*_mean_ = 20). Experimental data for the six fractured 0.5 mm zirconia OVs (Z-0.5) and their respective number of cycles to fracture are indicated by open circles. Also shown are the estimated years in service, represented by vertical solid lines, based on an assumption of 1.2 million cycles corresponding to approximately five years (5Yrs) [30,31]. Notably, the factor that these six fractured experimental data points are situated around the lower bound of the 5Y-PSZ lifetime prediction provides some validation of our theoretical model.

Under cyclic fatigue loading (***F*** = 49 N) and utilizing the equations, material properties, and geometrical and dimensionless parameters described in Section 2.3, Figure 7 plots the number of cycles to fracture for the two ceramic OVs of varying thickness. The shaded gray area indicates the predicted lifetime of the zirconia OVs. The dashed line on the left represents the lower bound of the 90% confidence interval, corresponding to a slow crack growth velocity exponent of *N*_lower_ = 23, whereas the solid line on the right represents the mean lifetime for *N*_mean_ = 25. Similarly, the red shaded area indicates the predicted lifetime for lithium disilicate OVs, with the lower 90% confidence bound (dashed line) and mean (solid line) corresponding to *N*_lower_ = 17 and *N*_mean_ = 20, respectively. Open circles represent the experimentally determined number of cycles to radial fracture for the 0.5 mm zirconia OVs. As shown, the experimental data for the six fractured zirconia OVs are all scattered around the predicted lower bound for this material, providing strong support for the accuracy of our theoretical prediction.

Based on mechanical properties, 0.5 mm thick lithium disilicate OVs may survive up to 10^10^ cycles—far exceeding any human lifespan. In comparison, OVs made of 5Y-PSZ developed radial cracks even before 10^6^ cycles in a 5-year simulation. While 0.4 mm lithium disilicate veneers were estimated to last 10 years, their zirconia counterparts were predicted to fail before reaching 10^4^ cycles—well below the 5-year benchmark. However, with increased thickness (>0.7 mm), zirconia veneers may achieve longevity comparable to or even exceeding, that of lithium disilicate.

## 4. Discussion

Experimental results showed that only the thinnest zirconia group (Z-0.5) developed radial cracks under fatigue, while all lithium disilicate and thicker zirconia specimens remained intact. Computational analysis supported these findings, predicting early failure for 0.5 mm zirconia veneers but long-term durability for lithium disilicate and thicker zirconia restorations. An increasing layer thickness improves fracture resistance through two key mechanisms. First, it reduces flexure and membrane stresses under occlusal loads, following a squared relationship with thickness. Second, it lengthens the crack propagation path, particularly significant for radial cracks at the intaglio surface. This effect is especially relevant for 5Y-PSZ OVs, which are more susceptible to radial cracks at reduced thicknesses and may experience significant durability improvements with increased layer thickness. Compared to zirconia, lithium disilicate is more susceptible to long-term weakening due its higher slow crack growth rate, making it more prone to degradation [43]. Slow crack growth occurs when water molecules or other corrosive agents weaken the strained ionic/covalent bonds at the crack tip, leading to a bond breakage that holds the crack walls together [44]. However, slow crack growth is a gradual process that unfolds over time, often taking years before resulting in fracture [33]. A previous scoping review recommended an occlusal veneer thickness of 0.7 and 1.0 mm for various ceramic materials [3]. The present findings support this recommendation, emphasizing that the ceramic layer thickness depends on material selection and should not be reduced below 0.7 mm when using 5Y-PSZ or the transition layer of multilayered zirconia (between 4Y-PSZ and 5Y-PSZ) for OV fabrication.

To date, no studies have investigated OVs fabricated from the transition layer of gradient multilayered zirconia composed of 4Y- and 5Y-PSZ. In a recently published in vitro study, ultrathin OVs (0.3 mm in the fissures/0.6 mm at the cusps) fabricated from different layers of IPS e.max ZirCAD Prime (3Y, 5Y and transition layer, Ivoclar Vivadent) and IPS e.max ZirCAD MT (4Y-PSZ, Ivoclar Vivadent) were subjected to thermocycling (7500 cycles, 5–55 °C) and chewing simulation (1,200,000 cycles, 98N). OVs made of 3Y-TZP, 4Y-PSZ and the transition layer (between 3Y-TZP and 5Y-PSZ) showed favourable fracture resistance. One veneer made of 5Y-PSZ exhibited a chipping and was assessed as failure. Additionally, 7.8% of 5Y-PSZ veneers were rated as partial failures [12]. In the present study half of the ultrathin zirconia veneer specimens (6 out of 12, 0.5 mm) developed flexural radial cracks (>1 mm in size) after chewing simulation, indicating that positioning veneers within the transition layer between 4Y- and 5Y-PSZ does not enhance fracture resistance. Experimentally determining strength gradients in the transition zone presents a technical challenge, making the exact mechanical properties of this region difficult to ascertain. Since the veneer restorations were placed near the boundary of the 5Y-layer, the mechanical properties of 5Y-PSZ were used for the calculations in the present study.

A thorough understanding of failure mechanisms is crucial for optimizing the clinical application of gradient multilayered zirconia in conservative restorative dentistry. Compared to monolithic zirconia, multilayered zirconia exhibits more complex failure mechanisms and appears to be more prone to radial cracks. This increased susceptibility may be attributed to differences in material composition and mechanical properties across layers, interlayering interfaces and flaw propagation across different layers [14]. Residual stress can develop within the ceramic material during fabrication, leading to interlayer variations that create potential stress concentration zones [45]. Additionally, layer interfaces may act as sites for crack deflection or delamination, contributing to unpredictable failure patterns. Clinically, radial cracks are more dangerous as they remain beneath the surface and close upon unloaded, making them difficult to detect through visual inspection [46]. Beyond their mechanical implications, subsurface cracks may also serve as potential sites of “contamination” in the oral environment. Fluid penetration, bacterial colonization or the ingress of dietary acids through such cracks could compromise the adhesive interface or accelerate degradation of both ceramic and luting cement. Since the radial cracks observed in this study were confined to the intaglio surface, they do not provide an immediate communication pathway to the oral cavity. However, once initiated, such subsurface cracks may propagate toward the outer surface, weaken the adhesive bond or potentially contribute to secondary caries [47]. While no data currently confirm this contamination pathway in occlusal veneers, previous studies have shown that cracks in natural teeth can harbor extensive bacterial colonization [48,49], indicating a clinically relevant risk that warrants further investigation. Maintaining an adequate thickness (>0.7 mm) seems to be essential for the longevity of gradient multilayered zirconia OV restorations, especially given their high elastic modulus but relatively low flexural strength. This requirement is even more critical than in 3Y-TZP and 4Y-PSZ zirconia (higher strength) and lithium disilicate (lower modulus) [3,12]. To minimize the risk of radial crack propagation, it is important to preserve this minimum thickness during preparation and to avoid unintentional reductions during final adjustments of the OV.

Discrepancies in elastic modulus between ceramics and their supporting structures can significantly affect the material’s load-bearing capacity [36]. A previous study showed that the load-bearing capacity of lithium disilicate (872 N) is superior to that of 5Y-PSZ (715 N) and even comparable to 4Y-PSZ (864 N), when supported by a dentin-like resin composite. While ultra-translucent 5Y-PSZ zirconia has significantly lower fracture resistance compared to 4Y-PSZ, both zirconia materials possess a similar elastic modulus (~210 GPa), which is considerably higher than that of lithium disilicate (~95 GPa) and dental hard tissues (enamel: ~70 GPa, dentin: ~18 GPa) [36]. Comparable analyses have been applied to other dental materials exposed to repetitive occlusal loading, such as 3D-printed resins used for splints and orthodontic devices. In these materials, fractal dimension and texture analysis revealed that rigid resins maintain surface integrity under compression, whereas flexible resins exhibited pronounced structural degradation and crack formation. Although their elastic moduli (~0.7–2.7 GPa) and fracture patterns differ markedly from ceramics, both materials classes exhibit occlusal force-induced microstructural changes, highlighting the value of fractal and textural analyses in fatigue assessment [50].

Several limitations of this study arise from its in vitro design. While in vitro testing is essential for assessing biomechanics, it cannot fully replicate the clinical complexity, including variable loading directions, chemical challenges and biofilm activity. To maintain consistency, typodont teeth and resin composite were used as substrates, which minimized biological variability (e.g., age, morphology, pre-existing defects and previous clinical history) and allowed standardized specimen preparation. Nonetheless, these substitutes do not entirely replicate the mechanical and structural properties of natural teeth. Furthermore, the theoretical analysis is restricted by the material constants embedded in the equations (e.g., elastic modulus, strength parameters), which are assumed to be homogeneous and isotropic. It was also assumed that the ceramic/resin cementcomposite interfaces are sufficiently strong, so that at the point of ceramic fracture, the entire system remains bonded together. Such implicit assumptions may not fully reflect the variability of the clinical conditions. Since previous investigations [12] on occlusal veneers reported fatigue cracks occurring exclusively in the 5Y-PSZ region, the veneers were positioned in the 4Y/5Y transition zone to explore whether comparable esthetics could be achieved while potentially enhancing fracture resistance. The mechanical properties, i.e., elastic modulus and flexural strength, of this zone were inferred based on its proximity to the 5Y layer, as direct strength measurement across the gradient zone remains technically challenging. This is because the elastic modulus of 5Y- and 4Y-PSZ is essentially the same, while the strength of a composition predominantly consisting of 5Y-PSZ is expected to approximate that of 5Y-PSZ, since fracture typically initiates in the weaker of the two materials. Nevertheless, this assumption introduces some uncertainty but was considered a conservative approach for evaluating worst-case performance. Fractographic analysis in this study was qualitative, aimed at verifying fracture origins and supporting the theoretical modeling approach. A statistical analysis, including comparisons across thicknesses and ceramic materials, was conducted in a preceding experimental study [32], which provided the basis for the present lifetime predictions. Finally, the study was limited to materials from a single manufacturer. While this ensured standardized processing and provided detailed data on material composition and properties required for the theoretical modeling, it may limit the generalizability of the findings. Future studies should therefore include materials from different manufacturers to enable for broader validation.

In clinical situations where high strength is essential and esthetic demands are moderate, such as in the molar region, monolithic zirconia (3Y-TZP or 4Y-PSZ) or lithium disilicate may be preferred over gradient multilayered zirconia. In esthetically demanding regions, OVs fabricated from multilayered zirconia can present a promising and viable restorative alternative. However, with reduced thickness, translucency differences between 3Y-TZP and 5Y-PSZ become progressively less pronounced. To ensure optimal performance and long-term durability, it is crucial to use multilayered zirconia with adequate layer thickness (>0.7 mm).

## 5. Conclusions

Gradient multilayered zirconia OVs placed in the transition zone between 4Y- and 5Y-PSZ exhibit a higher risk of fatigue-induced radial cracking at reduced thicknesses. In contrast, lithium disilicate veneers demonstrate superior fracture resistance under simulated oral conditions and may offer enhanced long-term performance. To optimize clinical outcomes, zirconia-based translucent OVs should maintain a minimum ceramic thickness of 0.7 mm.

## Data Availability

The original contributions presented in this study are included in the article. Further inquiries can be directed to the corresponding author.

## Abbreviations

The following abbreviations are used in this manuscript:

OV	Occlusal veneer
3Y-TZP	3 mol% Yttria-stabilized Tetragonal Zirconia Polycrystals
4Y-PSZ	4 mol% Yttria Partially Stabilized Zirconia
5Y-PSZ	5 mol% Yttria Partially Stabilized Zirconia
CAD	Computer-Aided Design
SEM	Scanning Electron Microscopy
BSE	Backscattered Electron
DC	Dual Curing
PM	PrograMill
Hz	Hertz
GPa	Gigapascal
MPa	Megapascal
N	Newton
µm	Micrometer
°C	Degrees Celsius
